# Study of an N6-methyladenosine- and ferroptosis-related prognostic model and the mechanisms underlying the molecular network in neuroblastoma based on multiple datasets

**DOI:** 10.1007/s12672-025-01975-9

**Published:** 2025-02-18

**Authors:** Jing Chu

**Affiliations:** Department of Pathology, Anhui Provincial Children’s Hospital, 39 Wangjiang East Road, Hefei, 230051 Anhui China

**Keywords:** Neuroblastoma, Bioinformatics, Ferroptosis, m^6^A, AKR1C1, Prognosis

## Abstract

Recent research highlights the pivotal role of N6-methyladenosine (m^6^A) modification and ferroptosis in the evolution of various cancers. This study aimed to establish a prognostic framework centered on genes associated with m^6^A and ferroptosis to enhance the accuracy of prognosis predictions for neuroblastoma (NB) patients, thereby improving targeted therapeutic strategies. Patient data, including expression profiles and clinical information from NB cases, were acquired from The Cancer Genome Atlas. Genes related to m^6^A modification and ferroptosis were identified, and those significant for prognosis were pinpointed using a combination of Cox regression analysis and the least absolute shrinkage and selection operator (LASSO) regression. For further validation, the study utilized external datasets GSE62564 and GSE85047. A prognostic index was computed for each NB patient, followed by analyses of immune cell infiltration and potential drug responsiveness based on the prognostic model. Additionally, enrichment analysis was conducted on the prognostic scores. These scores showed a strong association with the tumor immune environment and the efficacy of prevalent cancer therapies. Moreover, the model’s prognostic score emerged as an independent predictive marker for NB. This research succeeded in creating and confirming a prognostic model rooted in m^6^A and ferroptosis-linked genes, promising to enrich the prognostic understanding and treatment approaches for NB.

**Supplementary Information:**

The online version contains supplementary material available at 10.1007/s12672-025-01975-9.

## Background

Neuroblastoma (NB) is the most common extracranial solid tumor in children, accounting for approximately 8% of all childhood cancers and 15% of childhood cancer mortality [1]. International Neuroblastoma Risk Group (INRG) employs a classification system that utilizes multiple risk factors to stratify patients’ pretreatment. These factors encompass INRG imaging stage, age, and pathology (histology, differentiation, MYCN amplification, diploidy, and 11q aberration). By combining these factors, patients can be categorized into the following pre-treatment risk groups: very low, low, intermediate, and high [2]. Notably, high-risk NB constitutes about half of all NB instances. Despite the administration of comprehensive treatments-including conventional chemotherapy, surgical intervention, radiation therapy, intensified chemotherapy, differentiation treatments, and GD2-specific monoclonal antibody immunotherapy-recurrence is common among these patients, with less than 10% surviving beyond five years post-recurrence [3]. Thus, unveiling novel prognostic genetic markers and therapeutic targets for NB is of paramount importance. N6-methyladenosine (m^6^A), the most prevalent modification of mRNA in eukaryotes, takes place at the N6-position of adenosine. It is a dynamic mRNA modification controlled by the m^6^A “writer” complex, “eraser” enzymes, and “reader” proteins [4]. The enzyme IGF2BP3, associated with m^6^A, interacts with MYCN mRNA that bears the m6A modification, influencing the stability and production of MYCN mRNA. MYCN can also enhance IGF2BP3 expression by binding to its promoter, creating a reciprocal enhancement loop that drives the proliferation of NB cells [5]. Discovered in 2012, ferroptosis is distinguished by its reliance on iron and manifests through extensive lipid peroxidation at the molecular tier, alongside notable mitochondrial shrinkage observed at the ultrastructural scale [6]. In the context of NB, the regulation of cellular iron metabolism is notably influenced by MYCN through the upregulation of TFRC gene expression. This gene is responsible for encoding transferrin receptor 1, crucial for iron transport across the cellular membrane. This upregulation results in heightened sensitivity of NB cells to GPX4 inhibitors, thereby predisposing them to ferroptosis [7].

Further research into various cancers has revealed significant interplay between prime m^6^A modification enzymes and ferroptosis, influencing tumor behavior. For instance, NKAP has been shown to facilitate m6A-dependent processing of SLC7A11 mRNA, acting as a deterrent to ferroptosis within glioblastoma cells [8]. Similarly, bladder cancer studies have demonstrated that the m^6^A modification of NRF2 mRNA by WTAP not only spurs tumor cell proliferation but also mitigates erastin-prompted ferroptosis [9]. The domains of m^6^A modifications and ferroptosis have emerged as focal points in cancer research, given their acknowledged significance in tumor genesis and therapeutic intervention. Nonetheless, the intricacies of how ferroptosis and m6A modifications are intertwined with NB progression remain underexplored, specifically regarding the crucial m^6^A entities and ferroptosis-involved genes within NB. This investigation aims to bridge the knowledge gap by synergizing independent analyses of these two mechanisms. Through the creation of a visual bioinformatics model, it seeks to unveil potential prognostic markers and novel therapeutic avenues for NB, offering a richer, multidimensional exploration.

## Materials and methods

### Data acquisition

mRNA expression datasets for NB were retrieved from The Cancer Genome Atlas (TCGA) (accessible via https://portal.gdc.cancer.gov/), incorporating data from 159 NB tumor samples. The Series Matrix File GSE62564, hosting expression and survival data for 498 NB patients, was sourced from the Gene Expression Omnibus (GEO) at the National Center for Biotechnology Information (NCBI) (detailed at https://www.ncbi.nlm.nih.gov/geo/info/datasets.html), utilizing the annotation platform GPL11154. Additionally, Series Matrix File GSE85047, also from GEO, provided data on 269 NB patients, complete with expression profiles and survival details, using the annotation platform GPL5175. A comprehensive list of 484 genes associated with ferroptosis was compiled from the dedicated ferroptosis database (accessible at http://www.zhounan.org/ferrdb) [10], while a selection of 35 genes linked to m^6^A modification was curated based on existing scientific literature [11].

### Gene ontology (GO) and kyoto encyclopedia of genes and genomes (KEGG) analysis for functional annotation

To elucidate the functional impact of genes with altered expression profiles, an in-depth analysis was conducted utilizing the clusterProfiler package [12] within R. This analysis aimed to map out the intricate relationships among the genes’ functionalities by employing both GO and KEGG [13–15] frameworks. Pathways and functions emerging from GO and KEGG analyses that exhibited both *p*-values and q-values below 0.05 were deemed significantly enriched.

### Gene set enrichment analysis (GSEA)

Further examination into the distinct pathways influenced by m^6^A and ferroptosis was undertaken through GSEA [16]. This process involved leveraging annotated gene sets from the Molecular Signatures Database (MsigDB) [17] to pinpoint variations in signaling pathways across differentially expressed gene subsets. The focus was on gene sets that showed substantial enrichment with an adjusted *p*-value of less than 0.05, sorting them by their alignment scores. GSEA’s utility extends to categorizing tumors and elucidating their underlying biological context.

### Development of model and prognosis

In crafting the prognostic framework, genes exhibiting differential expression were pinpointed for inclusion. The model’s foundation was established through the application of least absolute shrinkage and selection operator (LASSO) regression [18], which facilitated the creation of a risk score formula for each patient by incorporating the expression values of selected genes. These scores were weighted according to coefficients derived from the regression model. Patients were then stratified into low- and high-risk categories based on median risk scores, serving as the delineation threshold. Survival differences between these groups were evaluated using the Kaplan–Meier method, with comparative analysis via a log-rank test. The prognostic relevance of the risk score was further validated through LASSO regression and segmented analysis. Finally, the predictive capability of the model was assessed through the generation of a receiver operating characteristic (ROC) curve.

### Development of nomogram

To construct a predictive nomogram, regression analysis was utilized, incorporating both the risk score and clinical manifestations of the disease. This process involved arranging scaled linear representations on a mutual plane, reflecting the relationships among predictive model variables based on predefined proportions. The predictive accuracy was enhanced by formulating a multivariate regression model. This model assigned scores to each determinant based on its relative contribution to the outcome, gauged by the size of its regression coefficient. Summing these individual scores yielded a comprehensive score for prediction.

### Analysis of immune infiltration

For the examination of immune cell presence within the tumor microenvironment, the Cell-type Identification by Estimating Relative Subsets of RNA Transcripts (CIBERSORT) approach [19] was employed. This method, founded on support vector regression, facilitates the nuanced deconvolution of immune cell subtype expression matrices. With 547 distinct markers, it discerns among 22 varieties of human immune cells, including various T and B cell lineages, plasma cells, and myeloid subsets. In this research, CIBERSORT was applied to deduce the proportional makeup of these 22 immune cell types from patient data, further exploring the interplay between gene expression levels and the prevalence of immune cells.

### Drug sensitivity analysis

The assessment of tumor sample responsiveness to chemotherapy was conducted using the pRRophetic package in R [20], leveraging the extensive Genomics of Drug Sensitivity in Cancer (GDSC) database [21]. This analysis determined the half-maximal inhibitory concentration (IC50) for each chemotherapy agent through regression techniques. The GDSC’s dataset served as a benchmark for validating the regression and predictive models via tenfold cross-validation. The analysis adhered to default parameters, including batch effect mitigation through ComBat and the averaging of expression levels for genes represented more than once.

### Gene set variation analysis (GSVA)

GSVA serves as a non-parametric and unsupervised approach for evaluating the enrichment of gene sets, effectively translating gene-level alterations to pathway-level implications. This method enables the assessment of biological function variations within samples [22]. For this investigation, gene sets were sourced from the MsigDB (version 7.0), with each set undergoing evaluation through the GSVA algorithm. This process aimed to identify shifts in biological functions across the analyzed samples.

### Preparation of human NB samples

Six children with NB, treated surgically at the Anhui Provincial Children’s Hospital, were selected for this study. Half were classified as high-risk and the other half as low risk. Tumor tissues, collected post-surgery, were stored at −80 °C. Only patients who had not received preoperative chemotherapy or radiotherapy were included.

### Cell culture and RNA interference

For experimental procedures, human NB cell lines, namely SK-N-BE2 (with MYCN amplification) and SH-SY5Y (without MYCN amplification), were acquired from Procell Life Science and Technology Co., Ltd. in Wuhan, China. Cells were periodically tested for mycoplasma contamination (C0297, Beyotime, Shanghai, China).

Lipofectamine™ 2000 Transfection Reagent (11668019, Thermo, USA) was mixed with siRNA to transfect NB cells. The sequences of the si-AKR1C1 were as follows:

- AKR1C1 si-1: (sense (5′–3′): CCACCAAAUUGGCAAUUGATT,
- antisense (5′–3′): UCAAUUGCCAAUUUGGUGGTT), and
- AKR1C1 si-2: (sense (5′–3′): GGCCGUGGAGAAGUGUAAATT,
- antisense (5′–3′): UUUACACUUCUCCACGGCCTT).

### RNA extraction and Reverse transcription-quantitative PCR (RT-qPCR)

The method was described in previously published paper [23]. Primer sequences utilized in the RT-qPCR are detailed in Supplementary file 1. The mean and standard error for each point were calculated for each sample in three separate reactions. The relative levels of mRNA transcripts were normalized to the control GAPDH. Relative gene expression was quantified using the GraphPad Prism 5.0 software.

### Western blot

The cells were lyzed in a RIPA lysis buffer containing protease inhibitors (P0013, Beyotime Biotechnology, Shanghai, China). The concentration of protein was evaluated by using the BCA Protein Assay Kit (Thermo, 23225). Western blot analysis was performed according to previous experimental procedures [24]. Primary antibodies included AKR1C1 (GeneTex, GTX105620, diluted at 1:1000), and β-Actin (Cell Signaling, #4967, diluted at 1:1000) was used to normalize the quantity of the loaded samples.

### Cell counting kit-8 (CCK-8) assay

Transfected SK-N-BE2 cells (3 × 10^4^ cells/well) were seeded into 96-well plates, and then incubated for 0, 24, 48, and 72 h respectively. The 10 μL CCK-8 solution (BS350B, Biosharp, Hefei, China) was then added and incubated for 1 h at 37 ℃.The absorbance value was measured at 450 nm on a microplate reader, and the number of viable cells was calculated.

### Wound healing assay

Transfected SK-N-BE2 cells (2 × 10^6^ cells/well) were seeded in 6-well plates to reach 100% confluence. The monolayer cells were scraped using a 10 μL pipette tip, and then incubated for 24 h. Photos were taken at 0 and 24 h after scratching, with a magnification of 40 times, to observe the scratch area.

### Transwell invasion assay

Transwell plates were used in combination with Matrigel matrix to detect the invasion ability of cells. Transfected SK-N-BE2 cells (2 × 10^6^ cells/well) were injected into the upper chambers with 100 μL base medium, and the lower chambers were added into medium containing 10% FBS. After 48 h of culture, the chambers were taken out, then discarded the culture medium, washed it twice with PBS, fixed it with 4% paraformaldehyde for 15 min, dyed it with 0.1% crystal violet for 10 min, and counted the cells under the microscope.

### Detection of apoptosis by YO-PRO-1/PI staining

The Apoptosis and Necrosis Detection Kit with YO-PRO-1 and PI (C1075S, Beyotime, Shanghai, China) was used according to the manufacturer’s instructions. YP1/PI working solution was added to transfected SK-N-BE2 cells (1 × 10^5^ cells/well) were seeded in a 24-well plate, then cells were incubated at 37 °C for 15 min in the dark. After incubation, observe the fluorescence staining effect under a fluorescence microscope and take photos (YP1-stained positive cells are green fluorescence, Ex/Em = 491/509 nm; PI-stained positive cells are red fluorescence, Ex/Em = 535/617 nm). The percentage of apoptotic and necrotic cells was manually counted and calculated.

### Statistical analysis

In terms of statistical analysis, survival outcomes were visualized through Kaplan–Meier plots and evaluated via the log-rank test for comparative purposes. Cox proportional hazards model facilitated multivariable analysis. The entirety of the statistical assessments was conducted using R software (version 4.2), adhering to a two-tailed test criterion with a significance threshold set at *p* < 0.05.

## Results

### Exploration of ferroptosis- and m^6^A-related prognostic genes in the NB cohort

**Fig. 1:**
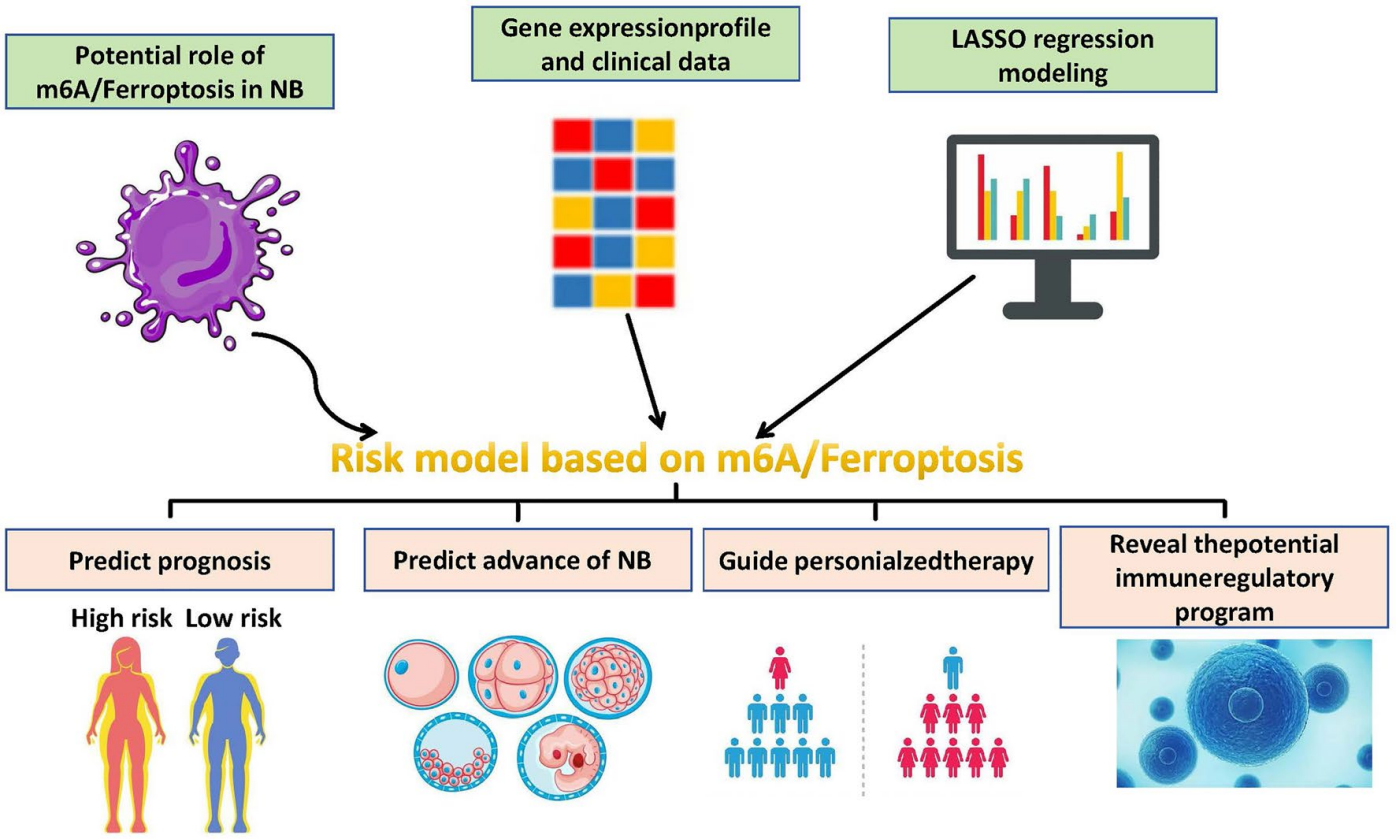
The flow chart describes the research idea and content of this study

This research undertook a comprehensive exploration, outlined in the study flowchart depicted in Fig. 1, into the prognostic significance of ferroptosis and m^6^A modification genes within a NB cohort. Utilizing TCGA for the acquisition of NB mRNA expression and clinical data, this study sourced 484 genes associated with ferroptosis from a dedicated database and identified 35 m^6^A-related genes through literature review. These genes were then independently analyzed to identify those with prognostic value using univariate Cox regression analysis.

**Fig. 2:**
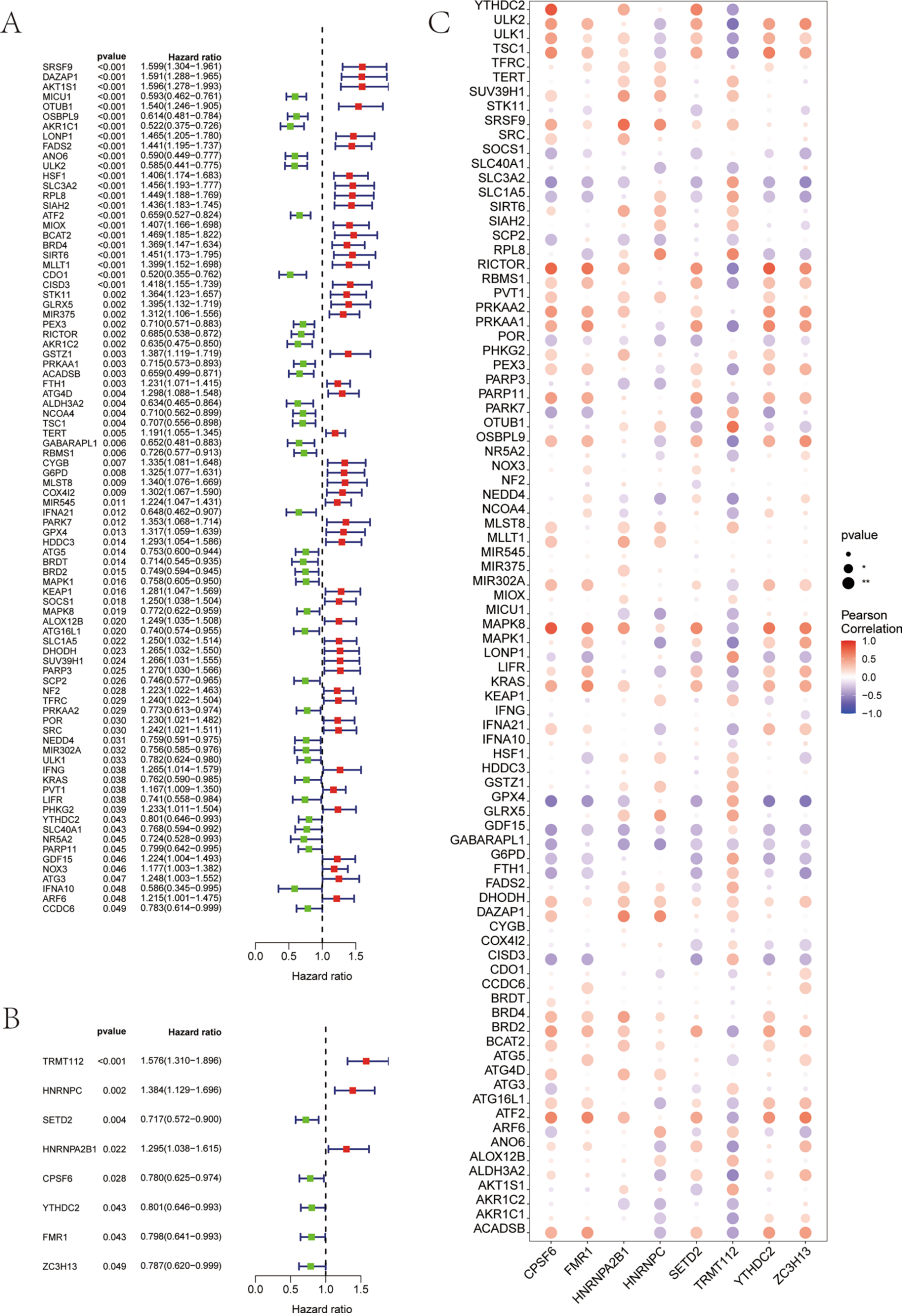
Ferroptosis- and m^6^A- related prognostic genes in the NB cohort. **A** 86 prognosis-related genes (*p* value <0.05) were screened out for ferroptosis. **B** 8 prognosis-related genes (*p* value <0.05) were screened out by m^6^A. **C** Correlation bubble chart of the 6 m^6^A molecules with the 86 ferroptosis-related genes (*P < 0.05, **P < 0.01)

The result showed that 86 ferroptosis-related prognostic genes were screened, and 49 of them were associated with a poor prognosis for NB; these included AKT1S1, SLC3A2, BCAT2, FTH1, GPX4, and DHODH (Fig. 2A, Supplementary file 2).

Eight m6A-related prognostic genes were screened, of which TRMT112, HNRNPC, and HNRNPA2B1 were determined to be risk factors for the prognosis of NB, whereas SETD2, CRSF6, YTHDC2, FMR1, and ZC3H13 were associated with a favorable prognosis for NB (Fig. 2B, Supplementary file 3). As demonstrated by the bubble plot (Fig. 2C), for instance, the m^6^A-associated gene HNRNPA2B1 showed a positive correlation with several ferroptosis-related genes such as SRSF9, SIRT6, PHKG2, and MAPK8. However, it exhibited a negative correlation with other ferroptosis-involved genes like SLC3A2, SCP2, and PARP3, highlighting the complex interplay between these two cellular mechanisms in the context of NB prognosis.

### Difference in prognosis between ferroptosis and m^6^A scores and enrichment analyses

**Fig. 3:**
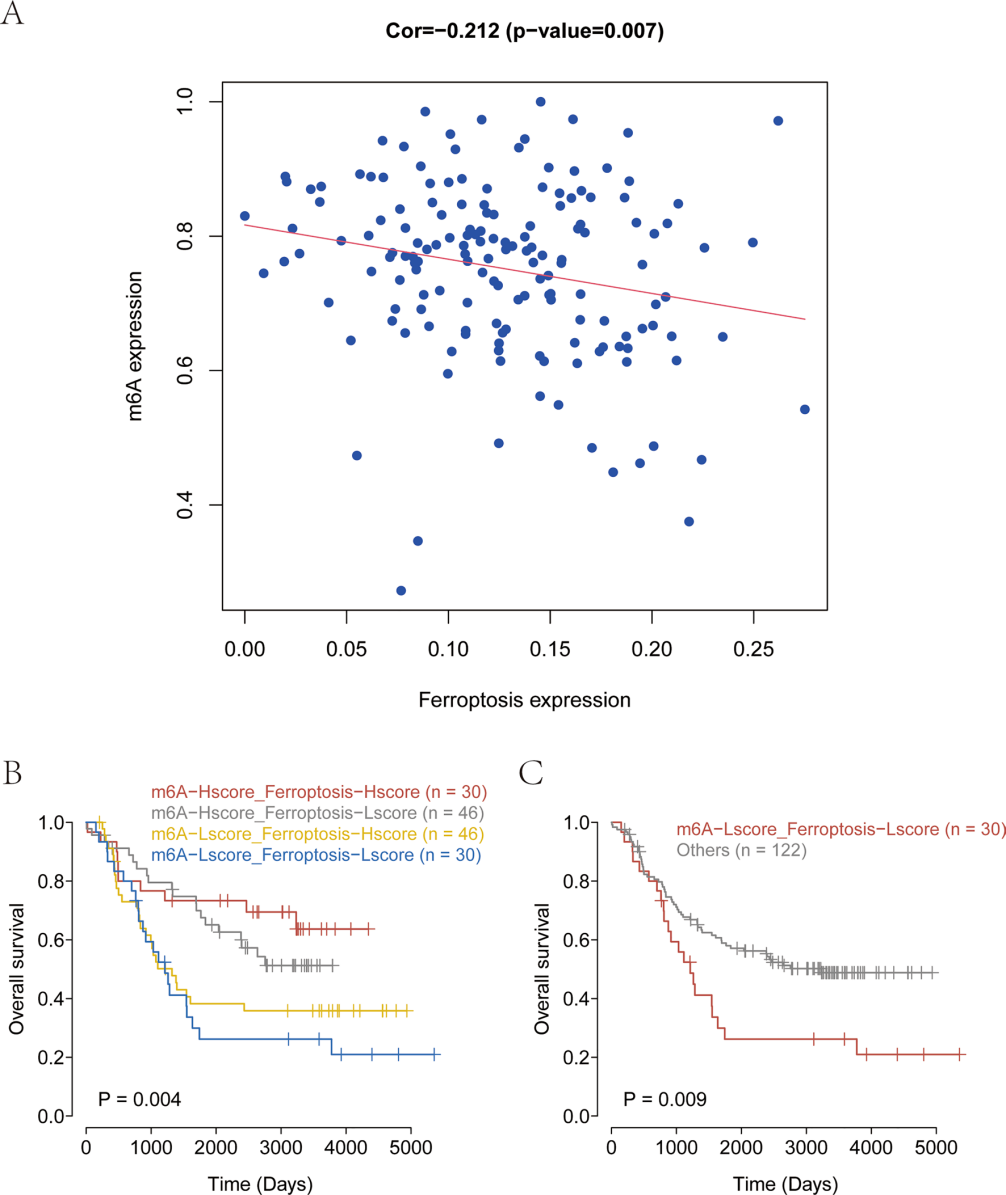
Difference in prognosis between ferroptosis and m^6^A scores. **A** A significant correlation between ferroptosis and m^6^A scores. **B** m^6^A–Lscore_Ferroptosis–Lscore had the worst prognosis. **C** m^6^A–Lscore_Ferroptosis–Lscore levels was associated with poor prognosis

To further investigate the crosstalk between ferroptosis and m6A, ferroptosis and m6A were rated using single sample GSEA (ssGSEA) based on the 86 ferroptosis-related prognostic genes and 8 m6A-related prognostic genes, and a significant correlation was found between ferroptosis and m6A scores (Fig. 3A). Next, the results of survival analysis based on high and low ferroptosis and m6A scores revealed that m6A-Lscore_Ferroptosis-Lscore had the worst prognosis (Fig. 3B). The data of the m6A-Lscore_Ferroptosis-Lscore group and the data of patients with NB in other groups were further used for the analysis of prognosis, and the results showed that m6A-Lscore_Ferroptosis-Lscore was associated with poor prognosis (Fig. 3C).

**Fig. 4:**
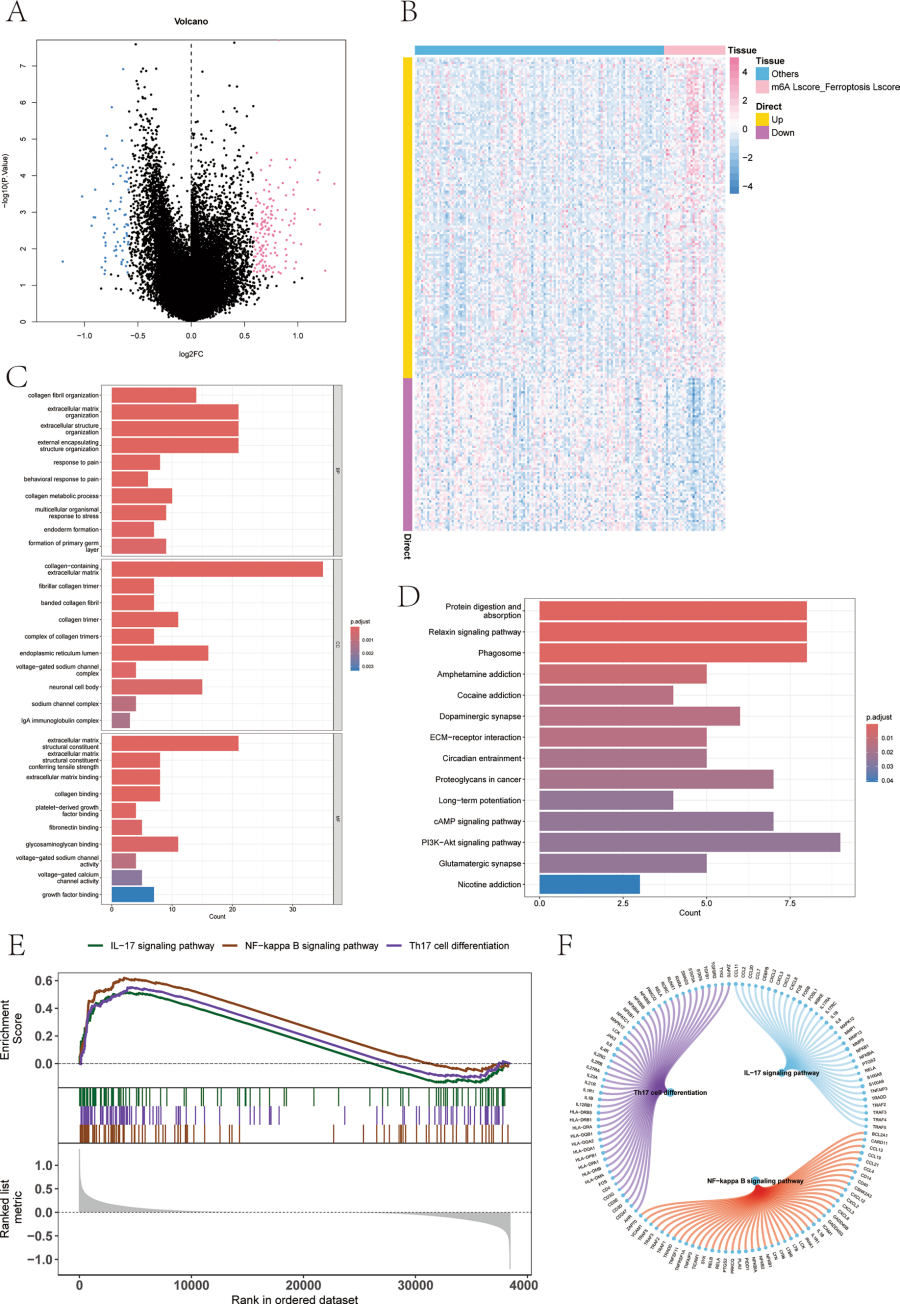
Differential and enrichment analyses of different ferroptosis and m^6^A score subgroups. **A**–**B** The differentially expressed genes between the m^6^A-Lscore_Ferroptosis-Lscore group and the others group. A total of 217 genes were screened. **C** GO results showed that the differentially genes were mainly enriched in pathways such as collagen fibril organization, extracellular matrix organization, and extracellular structure organization. **D** KEGG results showed that the differentially genes were mainly enriched in pathways such as Relaxin, cAMP, and PI3K-Akt signaling pathway. **E** GSEA results showed that the pathways involved were IL-17, NF-κB signaling pathway, and Th17 cell differentiation. **F** The network of molecular interactions among the pathways

Subsequently, the limma package was used to identify the differentially expressed genes between the m6A-Lscore_Ferroptosis-Lscore group and the others group, with the screening conditions being *p* < 0.05 and |logFC|> 0.585. In total, 217 differentially expressed genes were screened, of which expression levels of 147 were upregulated and those of 70 were downregulated (Fig. 4A–B, Supplementary file 4–6). These 217 differentially expressed genes were then subjected to pathway enrichment analysis, and the GO results demonstrated that the differentially expressed genes were mainly enriched in pathways such as collagen fibril organization, extracellular matrix organization, and extracellular structure organization (Fig. 4C).

The KEGG results indicated that the differentially expressed genes were mainly enriched in pathways such as the relaxin signaling pathway, cAMP signaling pathway, and PI3K–Akt signaling pathway (Fig. 4D).

Moreover, the GSEA results revealed that the pathways involved were the IL-17 signaling pathway, NF-κB signaling pathway, and Th17 cell differentiation (Fig. 4E). The network of molecular interactions among the pathways is shown in the following Fig. 4F. The subsequent figure illustrates the intricate network of molecular pathways, emphasizing the central role of PI3K in regulating various cellular processes like division, differentiation, and apoptosis. Previous research underscores the PI3K/Akt pathway’s contribution to NB cell proliferation and metastasis, notably through the induction of epithelial–mesenchymal transition (EMT) and the suppression of E-cadherin expression [25]. Moreover, the activation of NF-κB signaling has been implicated in cancer development, progression, and resistance to treatment. Strategies that inhibit the PI3K/Akt/NF-κB pathway by dephosphorylation have shown potential in curtailing NB cell proliferation, suggesting a viable therapeutic avenue [26].

### Development of prognostic model using the prognosis-related genes

**Fig. 5:**
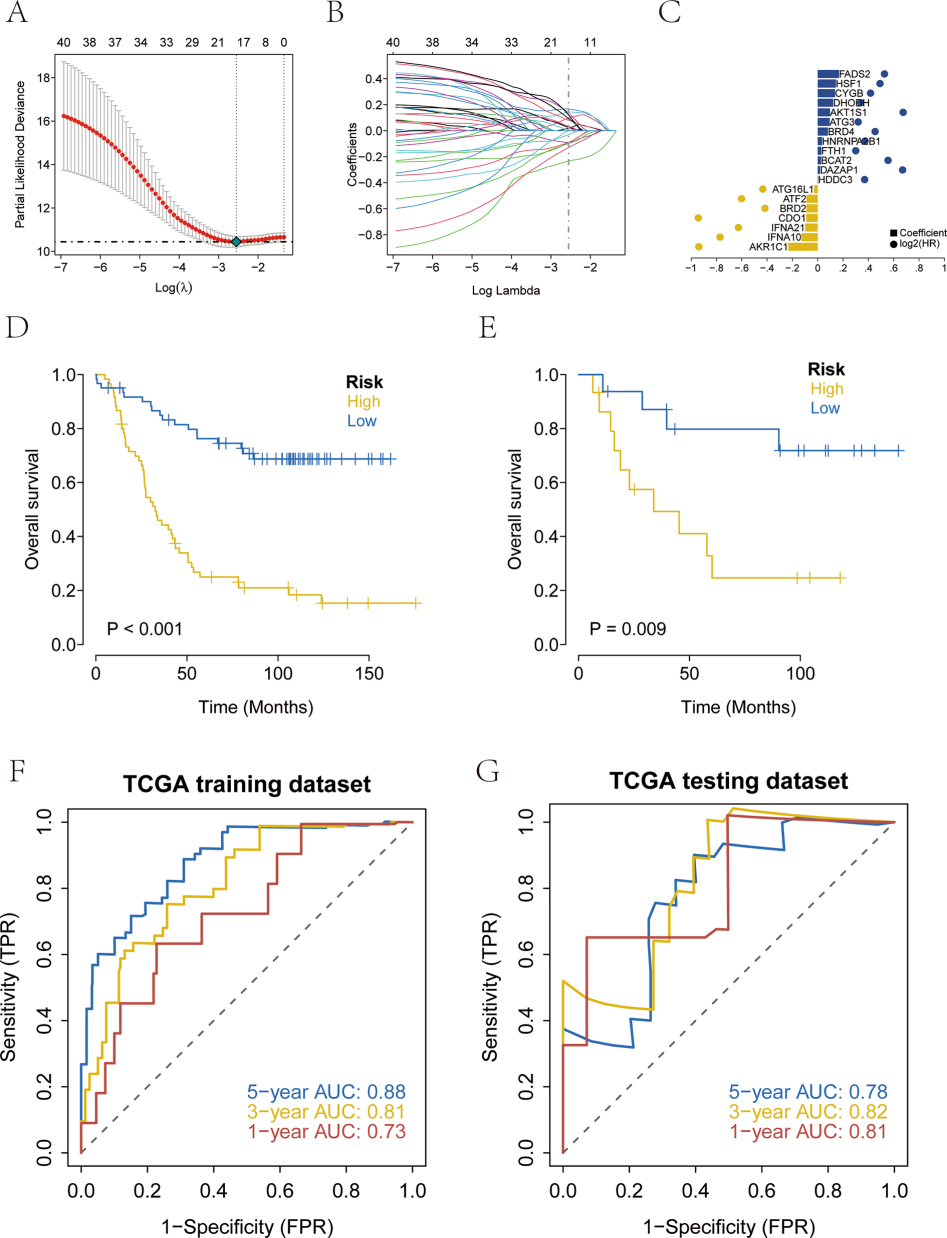
Construct a prognostic model using prognosis-related genes. **A** A coefficient profile plot was generated against the log (lambda) sequence. Selection of the optimal parameter (lambda) in the LASSO model for TCGA. **B** LASSO coefficient profiles of the 94 prognostic genes in TCGA-NB. **C** LASSO Coefficient HR. **D** Kaplan–Meier survival curve analysis in the high- and low-risk NB patients in the TCGA training subset. **E** Kaplan–Meier survival curve analysis in the high- and low-risk NB patients in the TCGA testing subset. **F** Time-dependent ROC curve for 1-year, 3-years, and 5-years prediction (TCGA training subset). **G** Time-dependent ROC curve for 1-year, 3-years, and 5-years prediction (TCGA testing subset)

To further identify the key genes among the 94 prognostic genes (86 ferroptosis-related genes and eight m6A-related genes), the clinical information of the patients with NB was collected, and LASSO regression for feature selection was used to screen the genes characterized in NB (Fig. 5A–C, Supplementary file 7). The patients were randomly divided into training and validation sets at a ratio of 4:1, and the best risk score corresponding to each sample was obtained using LASSO regression and used for subsequent analysis (Risk Score = AKR1C1 × (−0.23240044647716) + IFNA10 × (−0.129431042757006) + IFNA21 × (−0.0997239505039437) + CDO1 × (−0.091228023332947) + BRD2 × (−0.0899154393149668) + ATF2 × (−0.089026625789342) + ATG16L1 × (−0.0291623459581555) + HDDC3 × 0.003100203 + DAZAP1 × 0.018397558 + BCAT2 × 0.024994921 + FTH1 × 0.027984282 + HNRNPA2B1 × 0.033679011 + BRD4 × 0.079793098 + ATG3 × 0.094574777 + AKT1S1 × 0.09760961 + DHODH × 0.121449934 + CYGB × 0.13638992 + HSF1 × 0.143296272 + FADS2 × 0.168241836). The patients were categorized into high- and low-risk groups based on their risk scores, followed by Kaplan–Meier survival analysis. The high-risk group had a significantly lower overall survival (OS) than the low-risk group in both training and test sets (Fig. 5D–E). In addition, the ROC curves of both training and test sets suggested that the model has high validation accuracy (Fig. 5F–G).

### Validation of the robustness of the prognostic model using external datasets

**Fig. 6:**
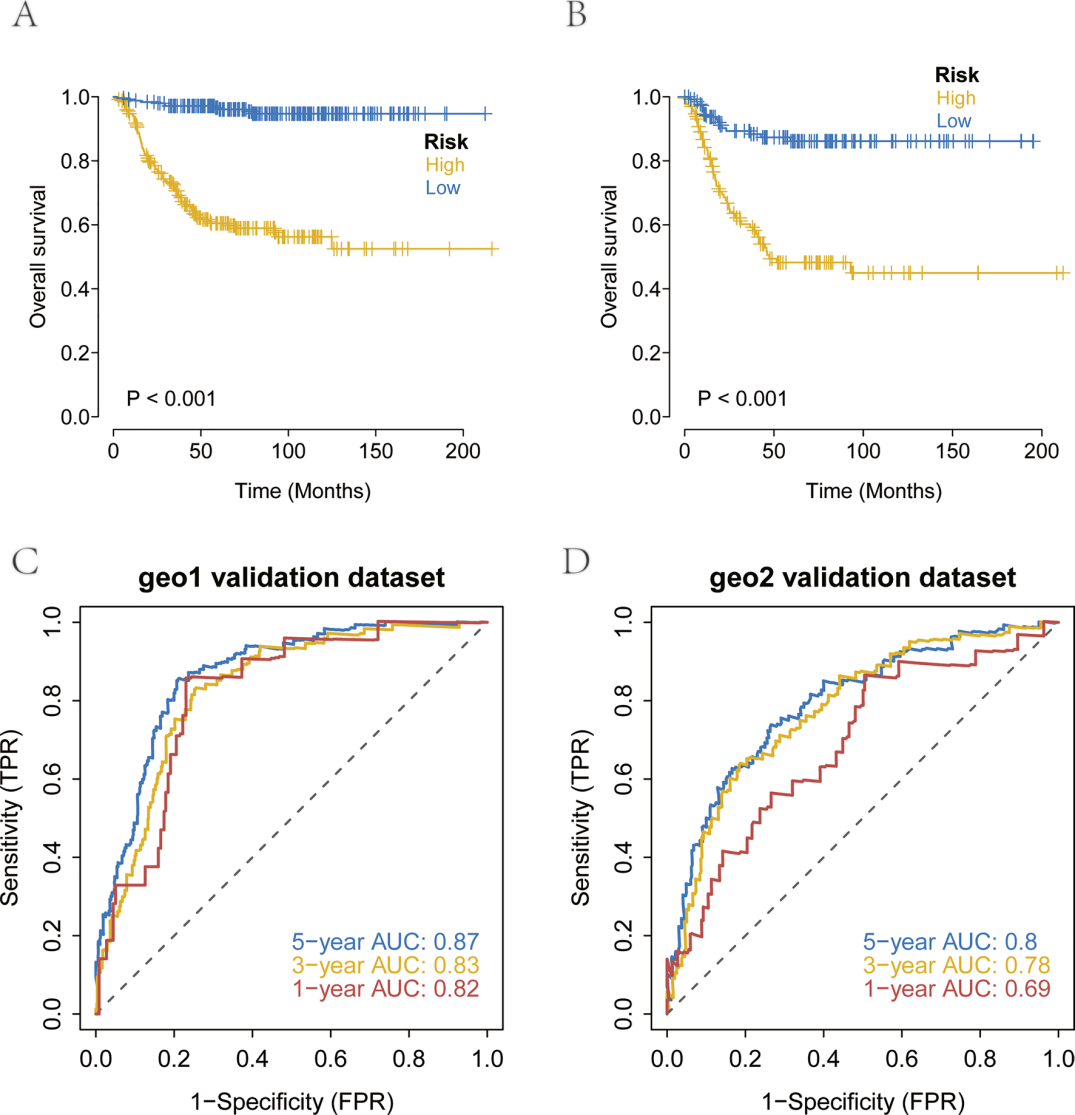
Validation of the robustness of the prognostic model using external datasets. **A** Kaplan–Meier survival curve analysis for GEO1 validation set. **B** Kaplan–Meier survival curve analysis for GEO2 validation set. **C** Survival ROC for external GEO1 validation dataset. **D** Survival ROC for external GEO2 validation dataset

To examine the prognostic model’s performance beyond the initial dataset, survival data for NB patients (from GSE62564 and GSE85047 datasets) were sourced from the GEO database. This step aimed to validate the model’s predictions on an external cohort by comparing survival outcomes between predicted high-risk and low-risk groups using Kaplan–Meier analysis. The external validation confirmed the model’s predictive strength, with high-risk patients exhibiting significantly shorter overall survival (OS) compared to their low-risk counterparts (Fig. 6A–B). Further evaluation through ROC analysis of the external datasets underscored the model’s predictive reliability for patient prognostication (Fig. 6C–D).

Furthermore, I conducted a comparative analysis of this prognostic model with previously reported ones and observed that it exhibited comparable or superior AUCs for OS prediction. The most notable aspect is that this prognostic model demonstrated superior reliability, as evidenced by its satisfactory and consistent performance in two external validation cohorts (Table 1).

**Table 1:** Comparison with previously reported prognostic models

Ref	Model	Training cohort	AUC (1-, 3-, 5-year OS)	Validation cohort one	AUC (1-, 3-, 5-year OS)	Validation cohort two	AUC (1-, 3-, 5-year OS)
Our model	19-gene	TCGA (n = 159)	0.88, 0.81, 0.73	GSE62564 (n = 498)	0.87, 0.83, 0.82	GSE85047 (n = 269)	0.8, 0.78, 0.69
Yu et al. [1]	13-gene	TARGET (n = 152)	0.767, 0.646, 0.617	GSE62564 (n = 492)	0.665, 0.641, 0.615	/	/
Wang et al. [28]	8-gene	TARGET (n = 148)	–, 0.825, –	GSE49710 (n = 498)	–, 0.778, –	/	/
Li et al. [12]	4-gene	GSE49710 (n = 498)	0.812, 0.845, 0.790	E-MTAB-8248 (n = 223)	0.835, 0.805, 0.781	/	/
Yang et al. [18]	9-gene	GSE49710 (n = 498)	–, 0.914, 0.911	TARGET (n = 144)	–, 0.727, 0.747	/	/
Wang et al. [6]	9-gene	TARGET (n = 114)	0.778, 0.788, 0.819	GSE49710 (n = 498)	0.711, 0.673, 0.718	/	/
Gupta et al. [29]	21-gene	TARGET (n = 243)	–, –, 0.840	E-MTAB-179 (n = 478)	–, –, 0.904	GSE85047 (n = 240)	–, –, 0.833
Li et al. [21]	3-gene	GSE49710 (n = 498)	0.838, 0.863, 0.839	E-MTAB-8248 (n = 223)	0.839, 0.840, 0.834	/	/
Ke et al. [22]	3-gene	E-MTAB-8248 (n = 223)	0.89, 0.82, 0.82,	GSE49710 (n = 498)	0.86, 0.85, 0.83	/	/
Zhou et al. [23]	5-gene	GSE49710 (n = 498)	0.84, 0.86, 0.89	E-MTAB-8248 (n = 223)	0.85, 0.78, 0.78	/	/

*AUC* area under the curve, *OS* overall survival

### Analysis of the correlation between the risk of morbidity and multiple clinical indicators and analysis of the independent prognostic factors

**Fig. 7:**
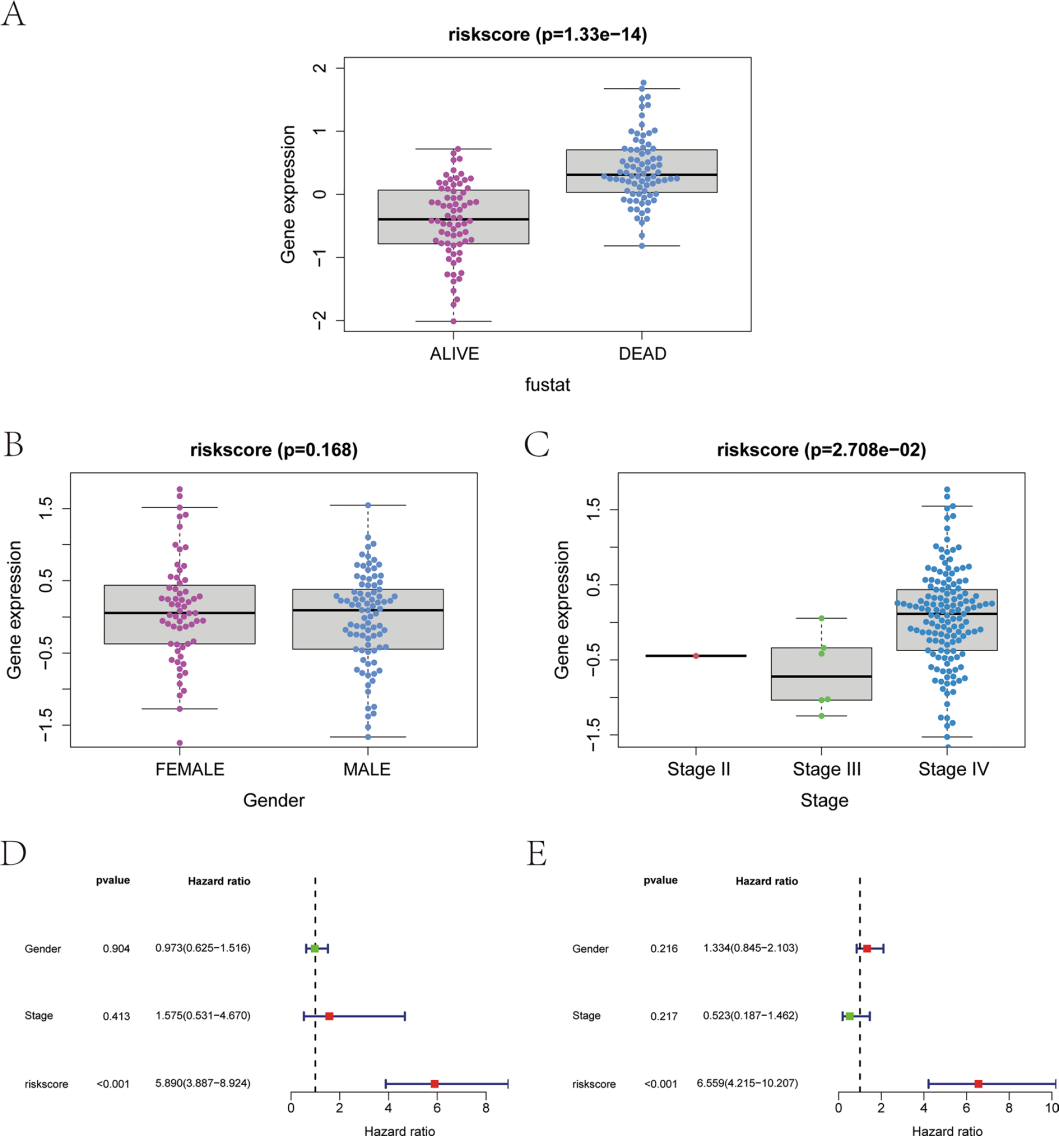
Correlation analysis between disease risk and multiple clinical indicators. **A** Relationship between fustat with risk scores. **B** Relationship between gender with risk scores. **C** Relationship between stage with risk scores. **D** Univariate Cox regression analysis. **E** Multifactorial Cox regression analysis

To delve deeper into the prognostic model’s clinical utility, the relationship between risk scores and various clinical parameters was analyzed. Patients’ risk scores were categorized based on significant clinical metrics, and differences across categories were visualized through box plots (Fig. 7A–C). A rank-sum test, focusing on survival status (Fustat) and cancer stage, revealed that risk scores varied significantly across these clinical indicators (*p*-value <0.05), affirming the model’s relevance in classifying NB patients. Additionally, the risk score’s role as an independent prognostic marker was investigated through univariate and multivariate analyses, establishing its standalone value in predicting outcomes for NB patients (Fig. 7D–E, Supplementary file 8–9).

**Fig. 8:**
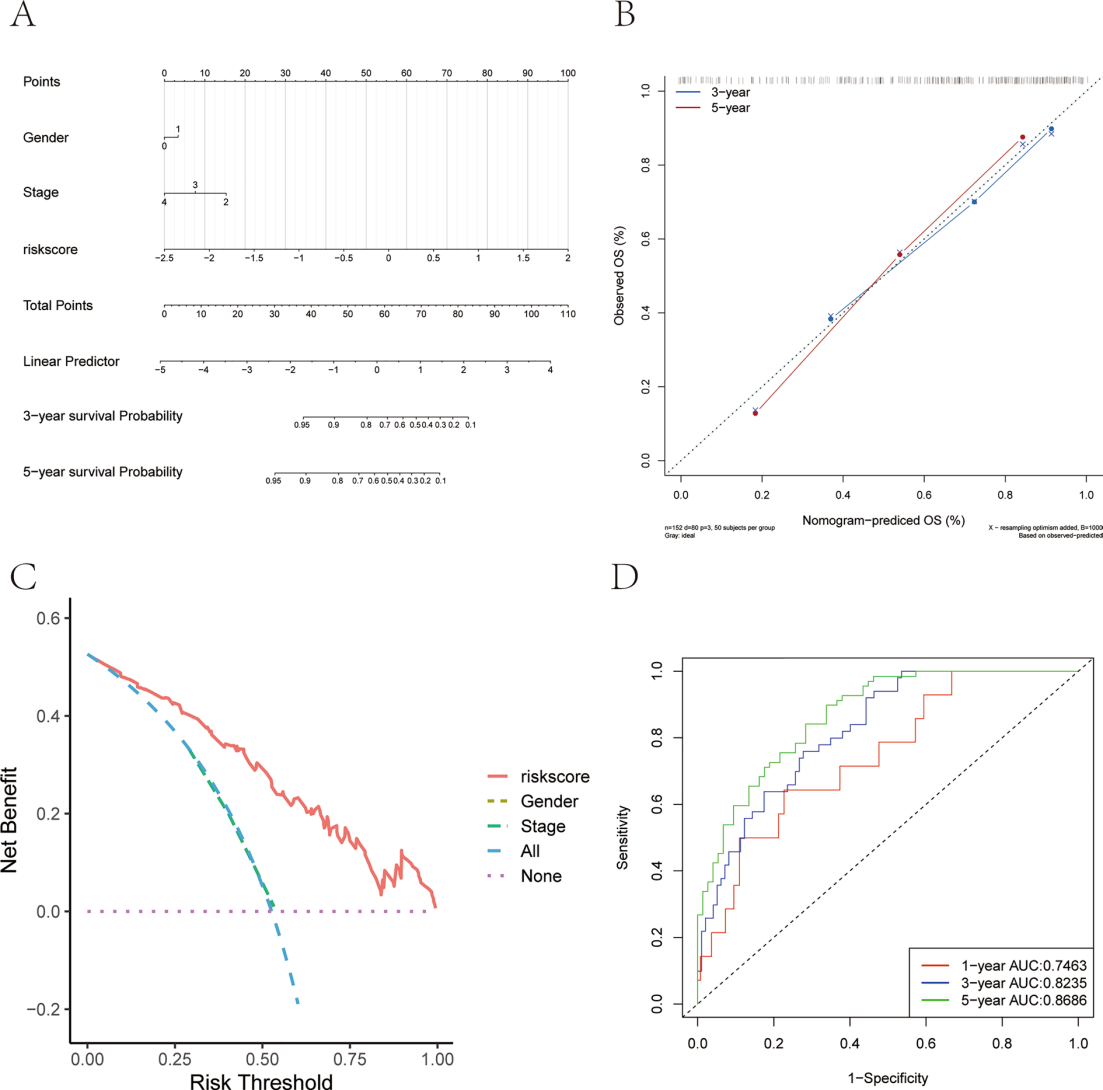
Risk of onset and independent prognosis analysis. **A** The nomogram for predicting the 3- and 5- years OS of NB patients. **B** The calibration curve of the nomogram for predicting 3- and 5-years OS of NB patients. **C** Decision curve analysis (DCA). **D** Time-dependent ROC curve for 1-year, 3-years, and 5-years prediction

The samples were further categorized into high- and low-risk groups using the median risk score, and the results of the regression analysis were presented using a nomogram. The results of this regression analysis demonstrated that the risk score had a substantial contribution to the process of scoring the nomogram prediction model for all samples of this study (Fig. 8A). Meanwhile, predictive analytics were also used for the 3- and 5-year periods, and the predicted and observed OS were found to be relatively consistent, indicating that the nomogram has good predictive efficacy (Fig. 8B–D).

### Relationship between the prognostic model and immune microenvironment

**Fig. 9:**
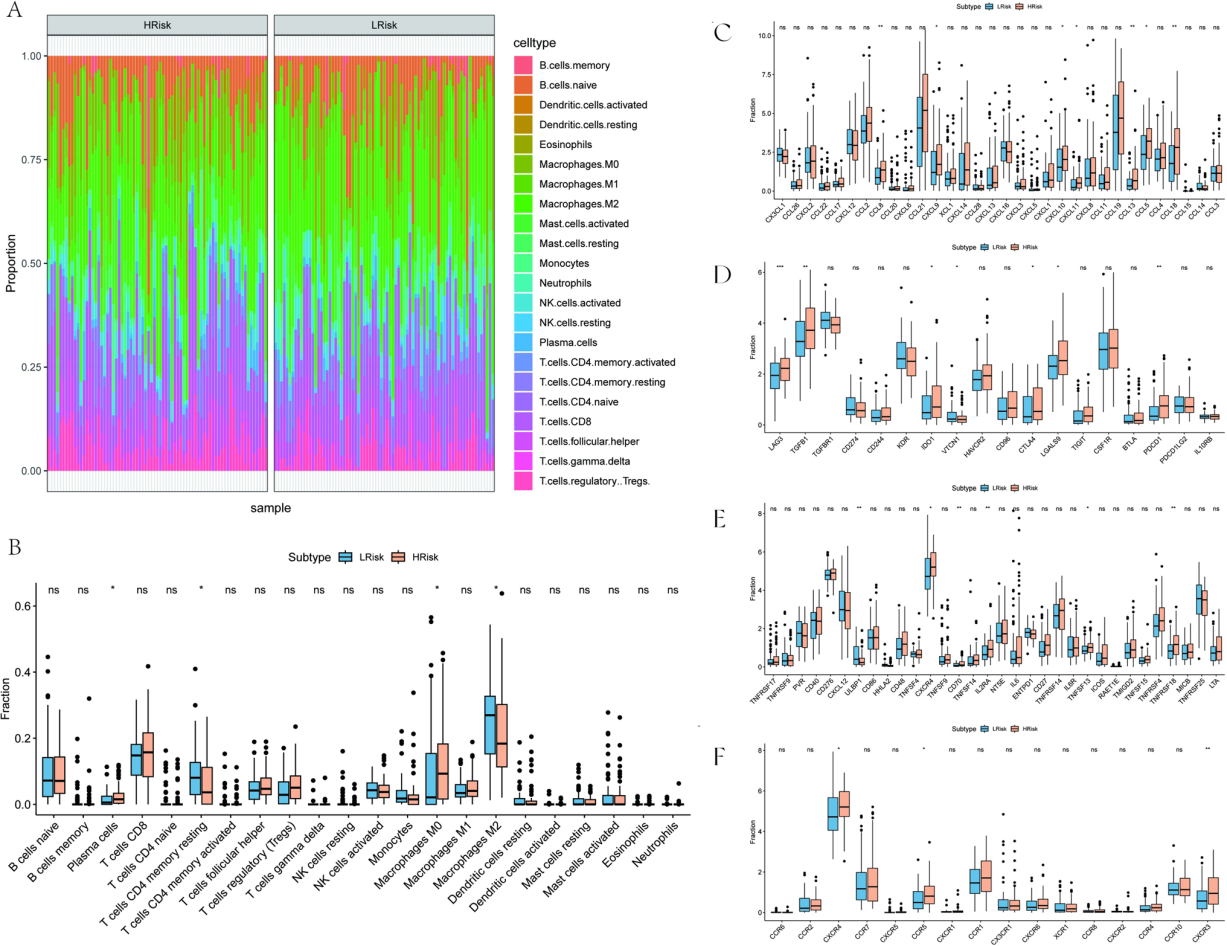
Relationship between the prognostic model and immune microenvironment. **A** The ratio of immune cells between the low- and high-risk groups. **B** Differences in the contents of immune cells were compared between the low- and high-risk groups. **C** Chemokine association. **D** Immuno-inhibitor association. **E** Immuno-stimulator association. **F** Receptor association

In the exploration of the tumor microenvironment (TME), which is a complex network composed of tumor-associated fibroblasts, immune cells, the extracellular matrix, growth and inflammatory factors, unique physicochemical properties, and cancer cells themselves, its pivotal role in shaping tumor diagnosis, progression, survival outcomes, and responsiveness to chemotherapy was emphasized. This study ventured into examining how risk scores correlate with tumor immune cell infiltration within the NB context, aiming to unearth the underlying molecular dynamics influencing NB advancement. Figure 9A illustrates the distribution of immune cells between groups stratified by risk, highlighting significant variances in immune cell composition.

The comparative analysis identified notable differences in immune cell populations between the low- and high-risk cohorts. Specifically, the high-risk group exhibited a pronounced depletion in resting CD4+ memory T cells and M2 macrophages, contrasted with an increase in plasma cells and M0 macrophages (Fig. 9B). The investigation extended to immunoregulatory genes, uncovering a significant upsurge in immune-related chemokines such as chemokine CCL8, and several C-X-C motif chemokine ligands (CXCL9, CXCL10, CXCL11) as well as CCL13, CCL5, and CCL18 within the high-risk group (Fig. 9C). Moreover, the high-risk group was characterized by heightened expression of immunosuppressive entities, including LAG3, TGFβ1, IDO1, CTLA4, LGALS9, and PDCD1 (Fig. 9D). Additionally, a surge in immunostimulatory molecules was observed, with elevated levels of ULBP1, CXCR4, CD70, IL2RA, TNFSF13, and TNFRSF18 being notable in the high-risk group (Fig. 9E). The study also pointed out significantly higher expressions of immunoreceptors, including CXCR4, CCR5, and CXCR3, in the high-risk group, indicating a complex interplay of immune responses associated with NB progression risk (Fig. 9F).

### Investigation of the clinical predictive value of the model based on a multi-omics study

**Fig. 10:**
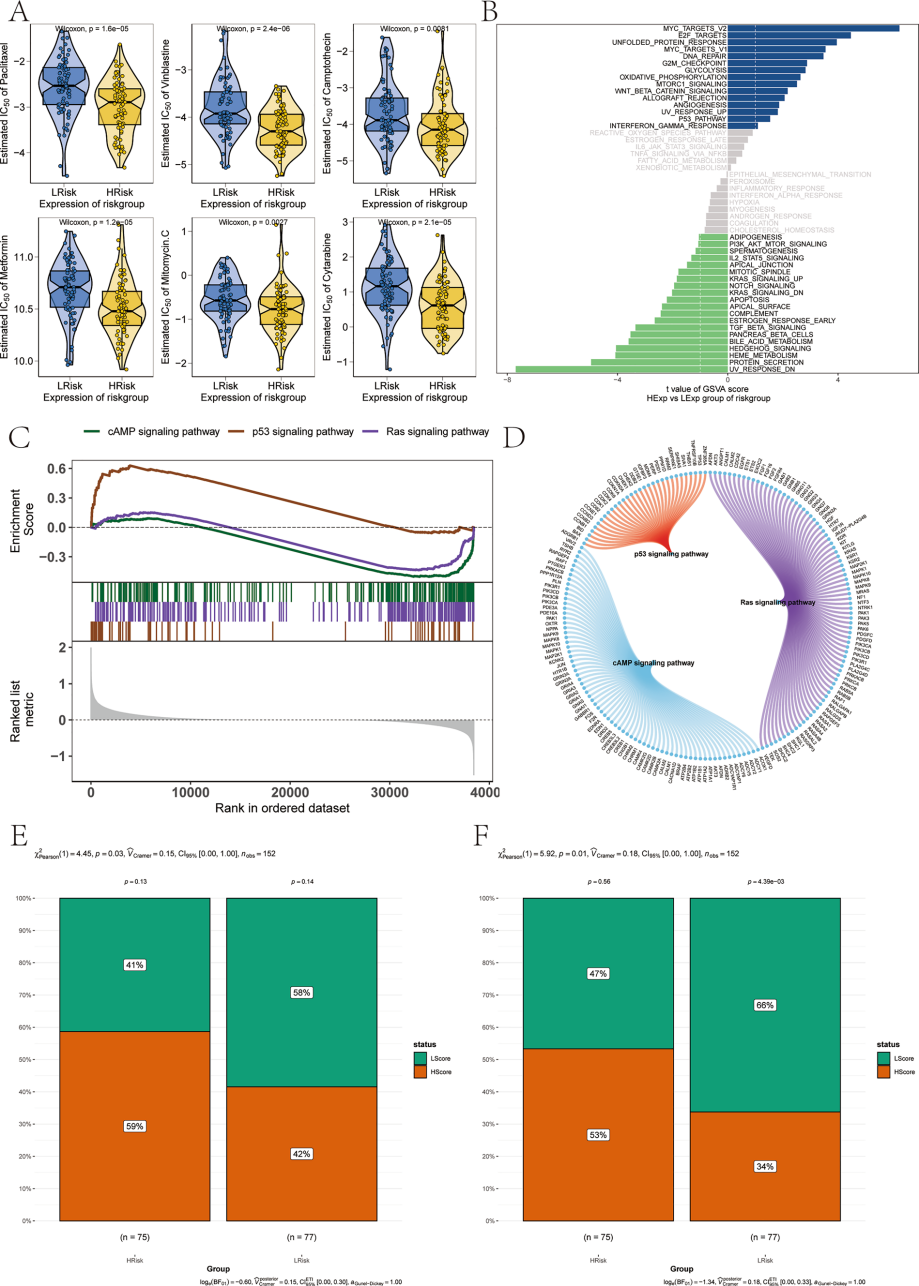
The clinical predictive value of the model based on a multi-omics study. **A** The difference on the therapeutic sensitivities of six chemotherapy drugs. **B** GSVA results showed that the differential pathways between the two groups of patients were mainly enriched in signaling pathways such as MYC_TARGETS_V2、E2F_TARGETS、WNT_BETA_CATENIN_SIGNALING pathway. **C** GSEA results show that the involved pathways were cAMP, p53, and Ras signaling pathway. **D** The molecular interaction network between each pathway in GSEA. In the analysis of Dysfunction (**E**) and MDSC (**F**)

The integration of surgery and chemotherapy in the initial stages of NB treatment has been well-documented for its efficacy. To delve deeper into the relationship between risk scores and the chemotherapeutic response, the ‘pRRophetic’ R package was employed, utilizing the GDSC database to predict tumor sample sensitivity to standard chemotherapy agents. This investigation uncovered a marked correlation between risk scores and the responsiveness to drugs such as paclitaxel, vinblastine, camptothecin, metformin, mitomycin C, and cytarabine (Fig. 10A). Furthermore, to elucidate the underlying molecular pathways that could be influencing tumor behavior based on risk categorization, GSVA was conducted. This revealed predominant enrichment in pathways including MYC_TARGETS_V2, E2F_TARGETS, and WNT_BETA_CATENIN_SIGNALING across different risk groups (Fig. 10B). GSEA further identified significant involvement of the cAMP, p53, and Ras signaling pathways (Fig. 10C). The network of molecular interactions among the pathways is shown in the following Fig. 10D.

The investigation extended to assessing the response of high- and low-risk groups to anti-tumor immunotherapy, specifically examining the Exclusion score. This score, indicative of the extent to which immune cells are barred from tumor proximity or hindered in lesion penetration, sheds light on the immune system’s efficacy against tumors. Elevated Exclusion scores, suggesting immune cell exclusion or impaired access to the tumor site, typically signal a compromised immunological defense against tumor cells, potentially facilitating tumor growth and progression. This phenomenon underscores a scenario where tumor cells circumvent immune detection, thereby harboring implications for poorer prognostic outcomes due to immune escape and surveillance evasion mechanisms [23]. My model revealed that the high-risk group had higher Exclusion scores than the low-risk group (Fig. 10E). The analysis also highlighted the role of myeloid-derived suppressor cells (MDSCs), often regarded as pivotal actors within the TME for their capacity to shield cancer from immune attack, thereby impeding immunotherapeutic efforts and favoring tumor endurance [27]. The model pinpointed a significant increase in MDSC scores within the high-risk group compared to their low-risk counterparts (Fig. 10F). This collective evidence from immunological assessments suggests that the high-risk group, as defined by my model, may exhibit a diminished response to immunotherapy, highlighting the critical need for tailored therapeutic strategies to enhance treatment efficacy.

### Validation of 19‑prognostic model genes in NB

**Fig. 11:**
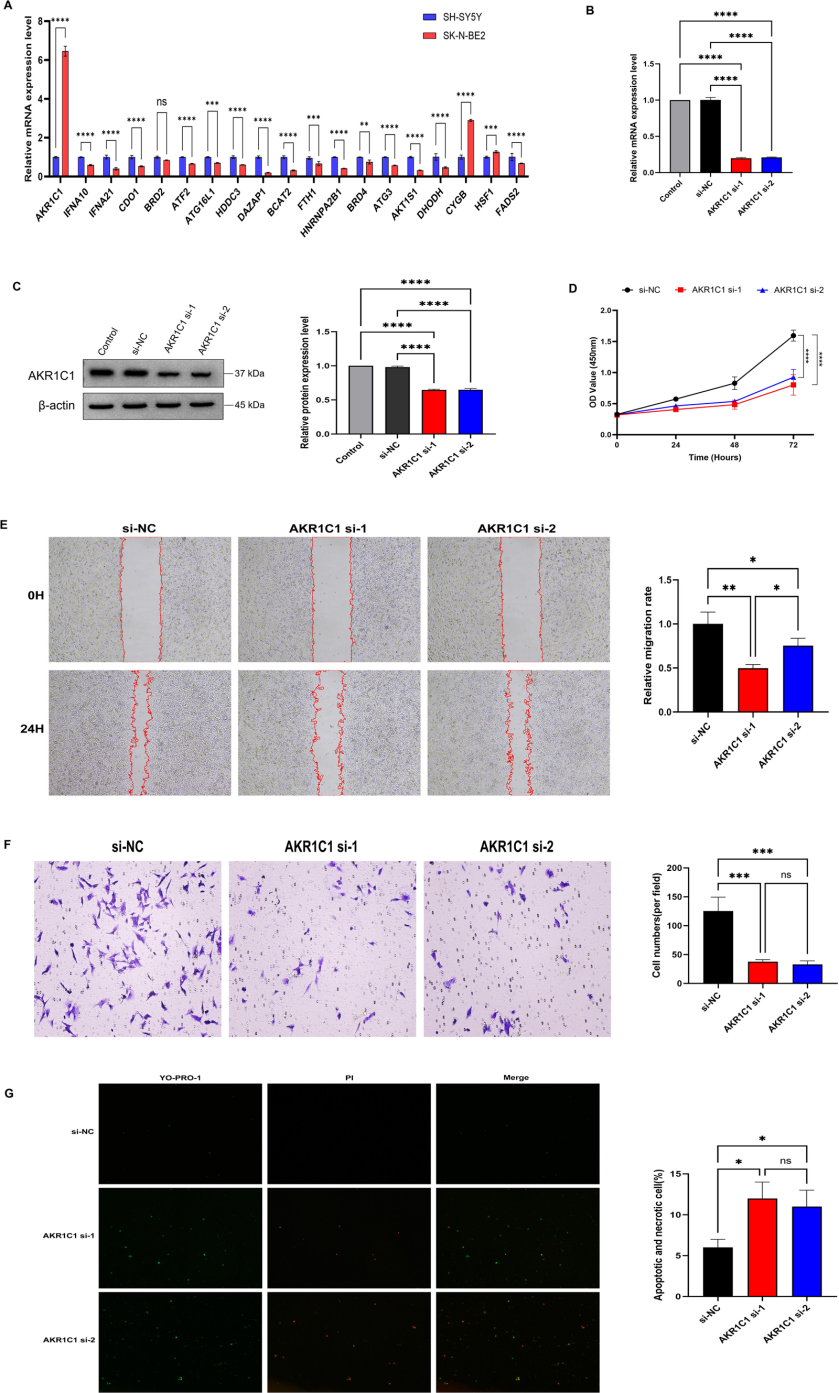
Cytological experiment in vitro. **A** The expression of the 19 prognostic genes in human NB tumor cell lines SK-N-BE2 (MYCN‑amplified) and SH-SY5Y (non‑MYCN amplified) by RT-qPCR. *P < 0.05, **P < 0.01, ***P < 0.001 vs. SH‑SY5Y cells. **B**–**C** RT-qPCR and Western Blot results showed that AKR1C1 was successfully knocked out by siRNA in SK-N-BE2 cells. NC, negative control. The viability, migration, invasion, apoptosis and necrosis rate of SK-N-BE2 cells after transfection of si-AKR1C1 #1/#2/NC were measured by CCK-8 (**D**), wound healing (**E**), transwell invasion (**F**) and YO-PRO-1/PI staining (**G**) assays, respectively. Data are presented as the mean ± standard deviation (SD). *p < 0.05, **p < 0.01, ***p < 0.001, ****p < 0.0001

The expression status of 19 genes in the prognostic model was detected in 6 cases of NB tumor samples via RT-qPCR, with equal representation from both high-risk (n = 3) and low-risk (n = 3) groups. The results indicated that, in comparison with the low-risk group, the mRNA expression levels of AKR1C1, BCAT2, HNRNPA2B1, ATG3, AKT1S1, CYGB, and FADS2 were significantly elevated in the high-risk group of NB; the mRNA expression levels of IFNA10, IFNA21, CDO1, ATF2, and ATG16L were significantly decreased in the high-risk group of NB; there were no statistically significant differences in the expression levels of BRD2, HDDC3, DAZAP1, FTH1, BRD4, DHODH, and HSF1 between the two groups of NB (Supplementary Fig. 1). The results of the cytological experiments indicated that elevated expression of AKR1C1, CYGB, and HSF1, contrasting with reduced expression levels of IFNA10, IFNA21, CDO1, BRD2, ATF2, ATG16L1, HDDC3, DAZAP1, BCAT2, FTH1, HNRNPA2B1, ATG3, AKT1S1, DHODH, and FADS2 in the MYCN-amplified NB cell line SK-N-BE2 in comparison to the non-MYCN amplified SH-SY5Y cell line (Fig. 11A). By comprehensively assessing the expression of 19 genes in NB tumor samples and cell lines, it was discovered that the mRNA expression level of AKR1C1 was significantly elevated in both high-risk NB groups and SK-N-BE2 (MYCN amplification NB cell line). Notably, AKR1C1 plays a critical role in mitigating ferroptosis by facilitating the enzymatic reduction of aldehydes and ketones into alcohols, thus impeding lipid peroxide formation [28]. To investigate the effect of AKR1C1 on the biological function of NB cells, si-AKR1C1 #1/ #2 and sh-NC were transfected into SK-N-BE2 cells. The expression of AKR1C1 mRNA and protein was significantly down-regulated in the AKR1C1 si-1 and AKR1C1 si-2 group, compared with that in the control (uninfected cells) and si-NC group (Fig. 11B, C). CCK-8 assay was further performed to examine the effect of AKR1C1 on the cell proliferation rate, with results indicating a significantly decreased proliferative capacity in the si-AKR1C1 group at 24, 48, and 72 h, compared to that in the si-NC group (Fig. 11D). The results of wound healing assay showed that the migration ability of SK-N-BE2 cells in the si-AKR1C1 groups was attenuated compared with that in the si-NC group (Fig. 11E). Transwell assay showed that the invasion ability of NB cells was inhibited by silencing AKR1C1 (Fig. 11F). In addition, apoptosis and necrosis analysis showed that the number of apoptotic and necrotic tumor cells in the AKR1C1 knockdown groups were significantly increased compared with the si-NC group (Fig. 11G). The above experimental data on cell function indicate that knockdown of AKR1C1 may defer the progression of NB by inhibiting the proliferation, migration, and invasion of tumor cells, and inducing tumor cell apoptosis.

## Discussion

In individuals diagnosed with high-risk NB, it is common to encounter stage 4 disease characterized by extensive metastatic spread, notably affecting the bone marrow, skeletal system, lymphatic nodes, and liver. The contemporary approach to treating such high-risk NB encompasses a multifaceted regimen including combined chemotherapeutic strategies, surgical intervention, radiation therapy, and immunological treatments. However, despite aggressive therapeutic efforts, a substantial proportion-about half-of those affected by high-risk NB experience recurrence, leading to dire consequences [29]. This underscores the urgent need for the identification of reliable biomarkers for the development of a prognostic framework, capable of evaluating the survival outcomes of NB patients accurately. The advent and rapid advancement of next-generation sequencing technologies have ushered in the utilization of computational methodologies for biomarker discovery and cancer research, marking a pivotal evolution in the realm of cancer systems biology. The trend towards employing integrative analyses that consider multiple phenotypes for the prediction of therapeutic responses in tumors has gained traction, demonstrating superiority over methods reliant on singular phenotypic or histological tumor characteristics [30].

My research unveiled an innovative prognostic framework grounded in the analysis of 19 distinct genes related to m^6^A modifications and ferroptosis processes (including AKR1C1, IFNA10, IFNA21, CDO1, BRD2, ATF2, ATG16L1, HDDC3, DAZAP1, BCAT2, FTH1, HNRNPA2B1, BRD4, ATG3, AKT1S1, DHODH, CYGB, HSF1, and FADS2), identified within the TCGA-NB project and further validated through two independent patient cohorts from the GEO database. Research indicates that AKR1C1 is overexpressed in osteosarcoma (OS) and correlates with poor prognosis in OS patients. In vivo and in vitro experiments reveal that the AKR1C1 inhibitor avasimibe inhibits OS cell proliferation and tumor growth by reducing the expression of AKR1C1 and FoxM1[31]. Likewise, AKR1C1 is overexpressed in cervical cancer (CC) tissues and is linked to the clinical characteristics of CC patients. In vitro studies suggest that AKR1C1 promotes the proliferation and invasion of CC cells by regulating the expression of TWIST1 and the AKT pathway [32]. In non-small cell lung cancer (NSCLC) cell lines, AKR1C1 expression is upregulated. Silencing AKR1C1 can inhibit the proliferation and migration of NSCLC cells and induce ferroptosis [33]. However, the biological function of AKR1C1 in NB remains unclear. RT-qPCR results from this study show that AKR1C1 mRNA expression is significantly upregulated in MYCN-amplified NB cell lines SK-N-BE2. Silencing AKR1C1 can inhibit the proliferation, migration, and invasion of SK-N-BE2 cells and induce apoptosis. Thus, AKR1C1 may serve as a specific marker and potential therapeutic target for NB patients.

The Bromodomain and Extra-Terminal (BET) protein family, comprising BRD2, BRD3, BRD4, and BRDT, plays a crucial role in the transcriptional regulation through recognition of epigenetic markers like N-acetylated lysine on histones, implicating them in the development and progression of various cancers [34]. Targeted inhibition of BET proteins has been shown to disrupt NB tumorigenesis by modulating MYCN expression [34]. FTH1, by regulating iron metabolism and the Fenton reaction, acts as a pivotal regulator in ferroptosis, with its deficiency leading to heightened ROS production and increased susceptibility of RAS-proficient NB N2A cells to ferroptosis [35]. The m^6^A mRNA reader hnRNPA2B1 enhances the stability of oncogenic mRNAs in an m^6^A-dependent manner, promoting the progression of cancer [36]. Elevated expression levels of DHODH have been identified as a marker of aggressive disease in NB, correlating with poorer survival metrics. The inhibition of DHODH in NB models via brequinar significantly curtails tumor growth and prolongs survival [37]. The proteins ATG16L1 and ATG3 are identified as critical to various stages of autophagy [38], suggesting that targeting the interactions among m^6^A modifications, autophagy, and ferroptosis may offer new avenues for the treatment of NB, potentially enhancing therapeutic outcomes.

Subsequently, I explored the relationship between the prognostic model and various clinical parameters, affirming its clinical predictive value through survival analyses and scrutinizing the OS rates across different patient groups as determined by the 19-gene prognostic model. Survival analysis, employing the Kaplan–Meier method, revealed that the OS rates for patients categorized within the high-risk group were significantly inferior to those in the low-risk group across both training and validation datasets. Moreover, ROC curve analysis validated the strong predictive capability of the model in forecasting patient prognosis. Both univariate and multivariate analyses confirmed the prognostic model’s risk score as an independent predictor for NB patient outcomes, with rank-sum testing revealing notable discrepancies in risk scores across different survival statuses and disease stages.

Exploring the connection between tumor risk scores and the degree of immune cell infiltration within tumors revealed variable levels of immune cell presence. Specifically, lower risk patients displayed higher concentrations of certain immune cells, like resting memory CD4 T cells and type M2 macrophages, while higher risk individuals exhibited increased numbers of plasma cells and undifferentiated macrophages (M0). M0 macrophages, which are in an undifferentiated state, possess the potential to evolve into either the M1 or M2 subtype depending on environmental signals. Research by Xu et al. [39] highlighted a predominance of M0 macrophages in tissues affected by pancreatic ductal adenocarcinoma compared to surrounding non-tumor tissue, associating a high presence of M0 macrophages with unfavorable patient prognoses. Analysis across four GEO gastric cancer datasets revealed three distinct subclasses based on immune cell composition, with Chen et al. [40] noting an association between increased survival rates and elevated levels of resting memory CD4+ T cells within one of these subclasses.

Chemokines play a critical role in modulating immune responses within the TME, influencing processes such as cell migration, differentiation, and infiltration into tissues. The interaction between chemokines such as CXCL9, CXCL10, CXCL11, and their receptor CXCR3 is known to affect immune cell behavior, potentially enhancing tumor growth and spread [41]. Conversely, high levels of other chemokines like CCL5, CCL8, CCL13, and CCL18 have been correlated with improved outcomes in bladder cancer patients [42]. This study observed that high-risk patient samples manifested elevated expressions of CXCL9, CXCL10, CXCL11, CCL8, CCL13, CCL5, CCL18, and the CXCR3 receptor.

Investigating the specific biological pathways that differentiate high- from low-risk groups via GSVA and GSEA revealed distinct patterns of gene expression enrichment. Pathways such as MYC_TARGETS_V2, E2F_TARGETS, and WNT_BETA_CATENIN_SIGNALING were predominantly activated in high-risk cases. Intriguingly, the use of retinoic acid (RA), a known antagonist of MYC_TARGETS_V2, is a standard treatment aimed at inducing tumor cell differentiation and inhibiting growth in high-risk NB patients [43]. However, activation of this pathway in high-risk NB may lead to RA therapy resistance. Additionally, RAS pathway mutations, commonly seen in aggressive and relapsed NB cases, further complicate the disease prognosis and treatment responses [44], suggesting a need for targeted therapeutic interventions.

Sensitivity analysis of the 19-gene model in relation to various chemotherapeutics indicated a significant correlation with drugs like Paclitaxel, Vinblastine, and Metformin, which are staples in NB treatment. Notably, targeted delivery of Paclitaxel using nanoparticles was shown to enhance drug concentration in tumor tissues significantly [45]. Metformin, beyond its use in diabetes management, has demonstrated potential in inhibiting NB cell proliferation through specific signaling pathways [46]. The efficacy of immunotherapy, based on the prognostic model, appears limited for high-risk groups due to lower levels of immune exclusion and MDSCs, suggesting alternative strategies might be more beneficial for these patients.

The intersection of m^6^A modifications, autophagy, and ferroptosis offers a promising area for novel cancer treatment avenues. The regulation of autophagy-dependent ferroptosis by m^6^A modifications in NB cells points to the potential of leveraging these mechanisms for therapeutic purposes. This understanding paves the way for the development of innovative treatments that induce tumor cell death through ferroptosis, facilitated by advancements in molecular and nanoparticle technology [47].

## Conclusion

This research endeavor undertook a comprehensive exploration of m^6^A modifications and ferroptosis-linked genes within NB, leveraging a suite of bioinformatic methodologies. Through meticulous analysis, a cohort of 19 genes was pinpointed, each bearing a significant correlation with patient prognosis. These discoveries facilitated the creation of a novel prognostic risk score model, designed to precisely evaluate the future outlook for individuals battling NB. To my knowledge, this initiative marks the inaugural effort to forge a prognostic framework intertwining m^6^A alterations with ferroptosis indicators for NB. The insights garnered here lay the groundwork for the discovery of new therapeutic avenues and prognostic markers. Nonetheless, the study is not without its constraints. Predominantly, the reliance on existing database information necessitates further empirical validation within a broader patient assembly to cement the model’s predictive reliability. Moreover, while the study is anchored in bioinformatics, additional empirical investigations are essential to elucidate the intricate biological mechanisms at play.

## Supplementary Information

Additional file 1.

Additional file 2.

Additional file 3.

Additional file 4.

Additional file 5.

Additional file 6.

Additional file 7.

Additional file 8.

Additional file 9.

Additional file 10.

Additional file 11.

Additional file 12.

## Data Availability

Publicly available datasets were analyzed in this study. This data can be found here: TCGA-NB (https://portal.gdc.cancer.gov/). GSE62564 (https://www.ncbi.nlm.nih.gov/geo/query/acc.cgi?acc=GSE62564). GSE85047 (https://www.ncbi.nlm.nih.gov/geo/query/acc.cgi?acc=GSE85047).
