# Supplementary material for: Study of an N6-methyladenosine- and ferroptosis-related prognostic model and the mechanisms underlying the molecular network in neuroblastoma based on multiple datasets
Source: Discov Oncol. 2025 Feb 18;16:200. doi: 10.1007/s12672-025-01975-9 (PMC11836251; doi:10.1007/s12672-025-01975-9)
Supplement: Supplementary file 1 — Additional file 1. [file 12672_2025_1975_MOESM1_ESM.docx]

**Supporting information**

Supplementary File 1: The list of primer sequences.

Supplementary File 2: 86 ferroptosis-related prognostic genes.

Supplementary File 3: 8 m6A-related prognostic genes.

217 differentially expressed genes (Supplementary File 4) between the m^6^A-Lscore_Ferroptosis-Lscore group and the others group, of which expression levels of 147 were upregulated (Supplementary File 5) and those of 70 were downregulated (Supplementary File 6).

Supplementary File 7: Lasso coefficient of 19‑prognostic model genes.

Supplementary File 8-9: Univariate and multivariate Cox regression analysis.

Supplementary File 10: Original images of western blot.

Supplementary Figure 1: Relative expression of 19 prognostic genes in clinical NB samples.
