# Supplementary figures and images for: Study of an N6-methyladenosine- and ferroptosis-related prognostic model and the mechanisms underlying the molecular network in neuroblastoma based on multiple datasets
Source: Discov Oncol. 2025 Feb 18;16:200. doi: 10.1007/s12672-025-01975-9 (PMC11836251; doi:10.1007/s12672-025-01975-9)

Original images of western blot

Figure 11C

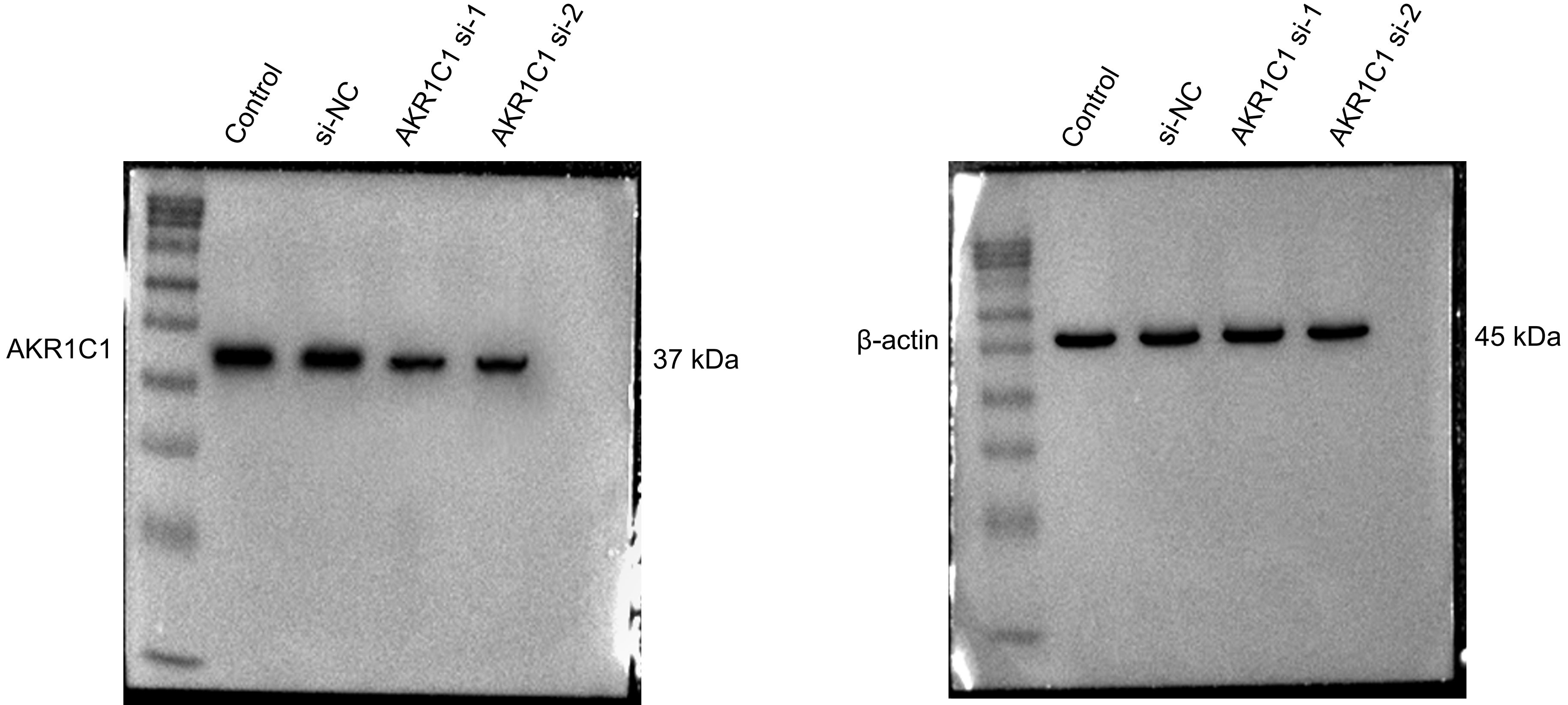

Supplement: Supplementary file 11 — Additional file 11. [file 12672_2025_1975_MOESM11_ESM.pdf]

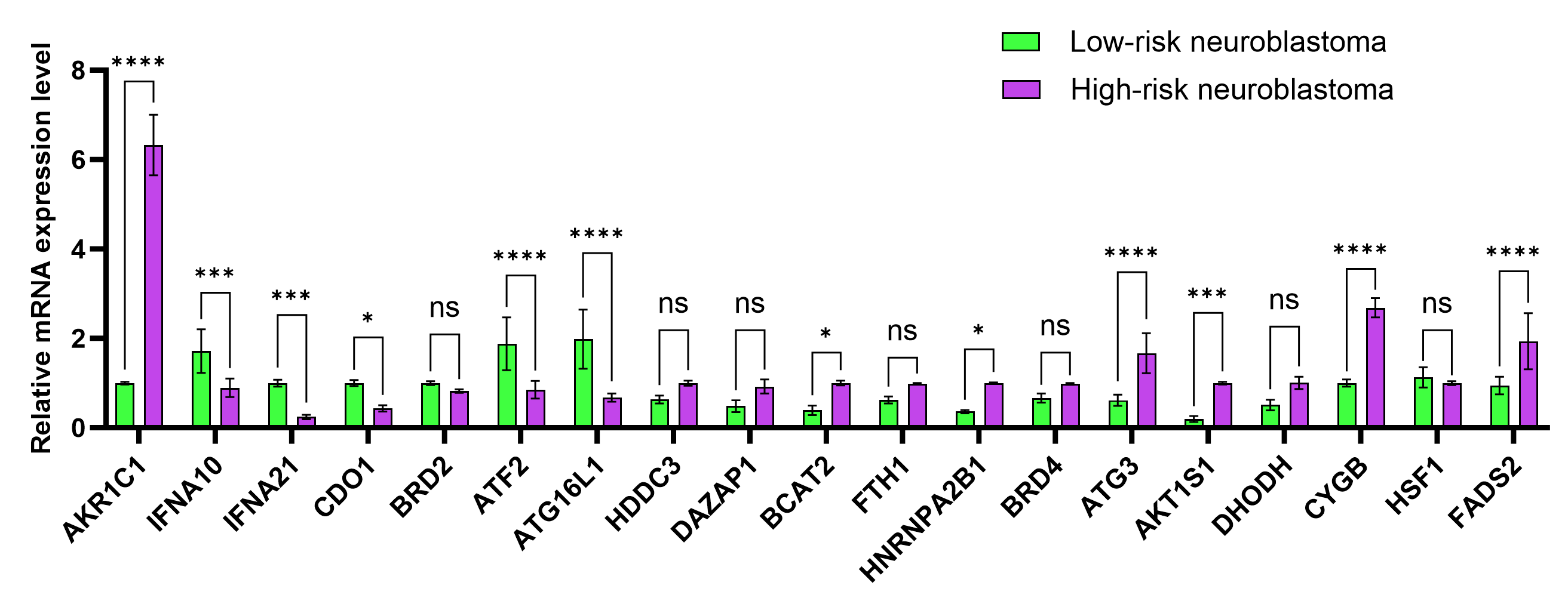

Supplement: Supplementary file 12 — Additional file 12. [file 12672_2025_1975_MOESM12_ESM.tif]
